# Mitochondrial DNA Variation Reveals a Sharp Genetic Break within the Distribution of the Blue Land Crab *Cardisoma guanhumi* in the Western Central Atlantic

**DOI:** 10.3390/molecules200815158

**Published:** 2015-08-19

**Authors:** Maria Rosimere Xavier Amaral, Marc Albrecht, Alan Shane McKinley, Adriana Márcia Ferreira de Carvalho, Severino Cavalcante de Sousa Junior, Fabio Mendonça Diniz

**Affiliations:** 1 Molecular Biology & Biotechnology Laboratory, EMBRAPA Meio-Norte, CP 01, Teresina, PI 64049-550, Brazil; E-Mails: rosiax2012@gmail.com (M.R.X.A.); dri_bio_uespi@yahoo.com.br (A.M.F.C.); 2 Department of Biology, University of Nebraska at Kearney, Kearney, NE 68849, USA; E-Mail: albrechtm@unk.edu; 3 National Park Service, 18001 Old Cutler Bay Rd Suite 419, Palmetto Bay, FL 33190, USA; E-Mail: mckinleyas@lopers.unk.edu; 4 Department of Zootechny, Universidade Federal do Piauí, Bom Jesus, PI 64900-000, Brazil; E-Mail: sevzoo@yahoo.com.br

**Keywords:** population structure, phylogeography, brachyuran crabs, sequencing, genetic diversity, control region, *mt*DNA

## Abstract

The blue land crab *Cardisoma guanhumi* is widely distributed throughout tropical and subtropical estuarine regions in the Western Central Atlantic (WCA). Patterns of population genetic structure and historical demographics of the species were assessed by *mt*DNA control region sequence analysis to examine the connectivity among five populations (*n* = 97) within the region for future conservation strategies and decision-making of fishery management. A total of 234 polymorphic nucleotides were revealed within the sequence region, which have defined 93 distinct haplotypes. No dominant *mt*DNA haplotypes were found but instead a distribution of a few low-frequency recurrent haplotypes with a large number of singletons. A NJ-tree and a median-joining haplotype network revealed two distinct clusters, corresponding to individuals from estuaries located along the Caribbean Sea and Brazilian waters, respectively. AMOVA and *F*_ST_ statistics supported the hypothesis that two main geographic regions exists. Phylogeographical discontinuity was further demonstrated by the Bayesian assignment analysis and a significant pattern of *isolation-by-distance*. Additionally, tests of neutral evolution and analysis of mismatch distribution indicate a complex demographic history in the WCA, which corresponds to bottleneck and subsequent population growth. Overall, a sharp genetic break between Caribbean and Brazilian populations raised concerns over the conservation status of the blue land crab.

## 1. Introduction

The blue land crab *Cardisoma guanhumi* Latreille 1825 (Decapoda: Brachyura: Gecarcinidae) is a nearly terrestrial species widely distributed throughout tropical and subtropical estuarine regions in the Western Central Atlantic, ranging from southeast Florida, Central America and the Bermudas, through the Gulf of Mexico and parts of the Caribbean Islands, to Brazil (from Ceará to São Paulo) [1,2,3,4,5]. Along the Brazilian coast, however, a distribution gap is observed between the states of Ceará and Amapá [6].

The species is intensively exploited as a food resource in many countries along the Atlantic coast of Central and South America, particularly Puerto Rico, the Bahamas, Honduras, Columbia, Venezuela, and Northeast Brazil [7,8,9,10], which has led to increase pressure on overfishing, and therefore, contributing to unsustainable fisheries throughout its distribution range. Consequently, crab fisheries have been declining along with species abundance in many countries [11,12,13], mainly due to unsustainable catch levels (*i.e.*, overexploitation) and ongoing habitat degradation. Therefore, the species is currently listed as “threatened, over-exploited, or threatened with overexploitation” in Brazil [14] and as “biologically vulnerable” by the Florida Fish and Wildlife Conservation Commission [15] in USA. This current fishing status has revealed the need for developing and implementing effective management strategies for the conservation of their wild stocks.

The knowledge of geographic patterns of population structure within the *C. guanhumi* distribution not only would help to obtain a deeper understanding of evolutionary process, but also is of paramount importance for management and conservation of the species. An understanding of its population genetic structure is critical in order to establish sustainable fishery policy priorities and management plans, as well as for defining effective conservation measures to maintain wild crab stocks at high levels of productivity [16,17,18].

Genetic structure of benthic organisms with a planktonic larval stage is dominantly determined by the probability of larval exchange among populations, and therefore, influenced by the dispersal ability of individuals and their larvae. Potential larval flows (gene exchange) between two putative populations, in turn, may be driven by biological (e.g., larval period, growth rate) and ecological factors (e.g., physical oceanographic processes, oceanic dispersal barriers) [19,20,21,22].

*C. guanhumi* females from the estuaries migrate to the shore and release eggs into saltwater. Once hatched, free swimming larvae molts through five larval stages that may last at least 42 days under laboratory conditions [1,3,23], when larvae have the potential to be transported to distant populations throughout ocean current processes. However, the freshwater plume from the Orinoco and Amazon Rivers, nearly 20% of the total global annual fresh-water discharge via rivers [24], along with the North Brazil Current (NBC) rings, may impose a zoogeographic barrier to the physical mechanisms of *C. guanhumi* larvae dispersal/retention. This, in turn, would respond to regional endemism patterns existing in this ocean region, as seen for many other species [25,26,27,28,29], and generally be attributed to the presence of cryptic species.

These possibly occurring evolutionary processes may be detected by the sequencing of the mitochondrial DNA (*mt*DNA) control region (CR), the most rapidly evolving and highly variable non-coding region in mitochondrial genome. The CR of the *mt*DNA has proved to be a useful tool to address evolutionary questions, e.g. the intra-specific population structure of many crab species [17,29,30,31,32,33,34].

In this investigation, patterns of population genetic structure and historical demographics of the blue land crab *Cardisoma guanhumi* from the Western Central Atlantic (WCA) were assessed by contiguous *mt*DNA sequences (a 112-bp 12S rRNA fragment and 598-bp of the 5′-end control region) to examine the connectivity among these populations within the WCA for future conservation strategies and decision-making of fishery management.

## 2. Results and Discussion

### 2.1. Sequence Diversity

**Figure 1:**
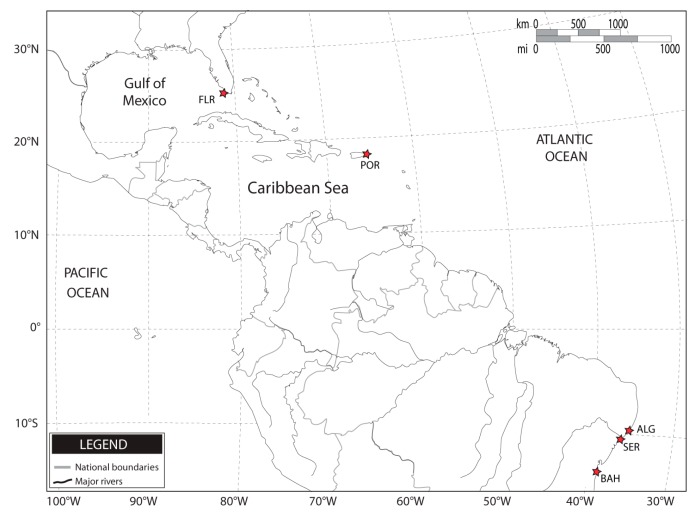
Sampling sites in the Western Central Atlantic region.

The analysis of mitochondrial DNA control region of *C. guanhumi* samples captured off the Western Central Atlantic (Figure 1) produced 97 sequences of approximately 710 bp each, which included 234 polymorphic nucleotide sites, 158 of which were phylogenetically informative and 76 singletons (Table 1). DNA sequences were blasted against the NCBI database (National Center for Biotechnology Information [35]), to confirm homology to accessions from the species of origin. A total of 93 distinct haplotypes were detected within localities. No dominant *mt*DNA haplotypes were found but instead a distribution of low-frequency recurrent haplotypes with a large number of singletons. Similar haplotypes were only detected in Brazilian mangroves (Sergipe and Bahia).

**Table 1:** Collection locations, sample size (N) and summary statistics of genetic variability for *C. guanhumi* populations along the Western Central Atlantic.

Locality	Code	Region	N	Nh	Np	Tv/Ts	M	Hd ± SD	π ± SD
Alagoas	ALA	Brazilian coast	15	15	72	0.1515	18.428 ± 8.663	1.000 ± 0.024	0.0266 ± 0.014
Bahia	BAH	Brazilian coast	15	15	71	0.2031	19.171 ± 8.999	0.971 ± 0.033	0.0277 ± 0.015
Sergipe	SER	Brazilian coast	22	21	90	0.1124	19.082 ± 8.787	0.996 ± 0.015	0.0276 ± 0.014
Florida	FLO	Caribbean Sea	21	21	140	0.1667	28.090 ± 12.809	1.000 ± 0.015	0.0406 ± 0.021
Puerto Rico	PUR	Caribbean Sea	24	24	158	0.1905	43.801± 19.686	1.000 ± 0.012	0.0633 ± 0.032

Nh, number of haplotype; Np, number of polymorphic sites; Tv/Ts, transversion/transition ratio; M, mean number of pairwise differences; Hd, haplotype diversity; π, nucleotide diversity; and standard deviation (SD).

The transversion/transition ratio was 0.1648, and base frequencies were *f*_A_ = 0.443, *f*_T_ = 0.313, *f*_C_ = 0.161, and *f*_G_ = 0.083. Genetic diversity indices (average ± standard deviation), haplotype diversity (Hd), and nucleotide diversity (π) are presented in Table 1. The mean haplotype diversity and nucleotide diversity in the Brazilian populations were 0.980 and 0.02, and in Caribbean populations were 1.00 and 0.05, respectively. Results from the substitution saturation analysis showed an *ISS* (0.09) much smaller than the critical *ISS* value (*ISS_C_* = 0.80), indicating that sequences are highly useful in phylogenetic reconstruction [36].

### 2.2. Phylogenetic and Network Analyses

**Figure 2:**
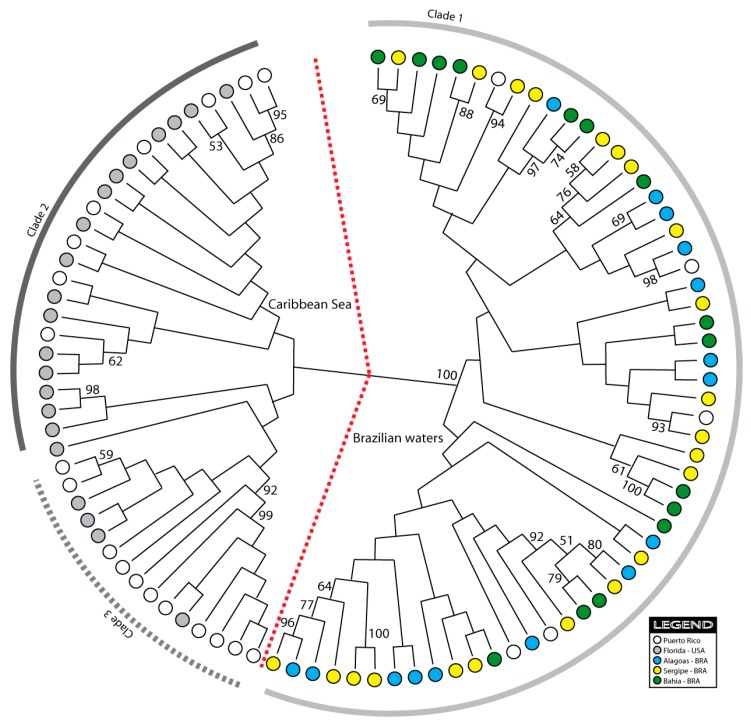
Neighbor-joining phylogenetic tree of the 93 *C. guanhumi* haplotypes collected from five sampling localities.

The neighbour-joining tree was constructed based on Kimura 2-Parameter distances of 93 individuals. In the tree, two very distinct clusters were observed, corresponding to the Caribbean Sea and the Brazilian coast (Figure 2). Only five Puerto Rican haplotypes were grouped within the Brazilian cluster.

**Figure 3:**
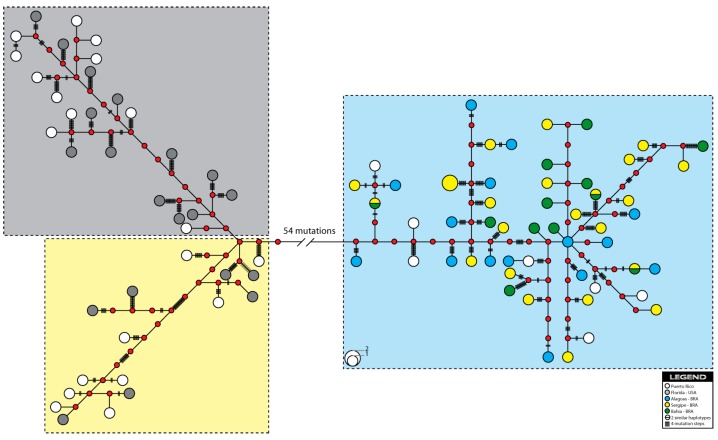
Median-joining network constructed for the *C. guanhumi* haplotypes in the Western Central Atlantic. Each circle represents one unique haplotype, with the area being proportional to the frequency of the haplotype in the five populations. Each haplotype is coloured according to the legend. Red circles indicate missing intermediate haplotypes that were not found in the analyzed individuals. Vertical bars on the line indicate the number of substitutions separating two haplotypes. A lack of vertical bars on the line connecting haplotypes indicates that a single substitution separates the two haplotypes.

The median joining network of *mt*DNA sequences for *C. guanhumi* showed the genealogical relationships among 93 haplotypes based on the least number of substitutions. The network consisted of two consecutively connected sub-networks corresponding to the two studied regions with most haplotypes connected by many mutation steps (Figure 3). No dominant haplotype was observed within this network as well. The two noticeable clusters were representing most of the Caribbean Sea and all Brazilian individuals, respectively.

### 2.3. Population Structure Analysis

The AMOVA of *C. guanhumi* *mt*DNA based on haplotype frequencies revealed that 51.26% of the genetic variation occurred within the populations, whereas 48.74% of the genetic variation occurred among populations (Table 2). The average fixation index (Φ_ST_ = 0.488, *p* = 0.00) departed significantly from zero, which indicated genetic structure was existed in *C.* *guanhumi* populations.

Further evidence of population genetic structuring within the five localities was revealed by *F*_ST_ analysis (Table 3). According to the *F*_ST_ values derived from the haplotype frequencies showed genetic differentiation between samples collected from the Caribbean Sea and from the Brazilian coast. No significant differentiation was observed among ALA, BAH, and SER at *p* < 0.05. However, BAH and ALA comparison revealed a slight differentiation at *p* < 0.10. Samples from FLO and PUR were also significantly different (*p* < 0.01).

Furthermore, all possible pairwise Da comparisons were high between Caribbean and Brazilian localities, and statistically significant. The largest genetic differences were found between the FLO and SER (Da: 48.19, *p* < 0.01).

**Table 2:** Analysis of molecular variation (AMOVA) of *C. guanhumi* *mt*DNA sequences.

Source of Variation	*df*	Sum of Squares	Variance Components	Percentage of Variation	Φ_ST_ (*p*-value)
Among populations	4	1097.095	13.529 Va	48.74	0.488 (*p* = 0.00)
Within populations	92	1309.117	14.229 Vb	51.26	
Total	96	2406.212	27.758		

**Table 3:** Corrected average pairwise difference Da (above diagonal) and *F*_ST_ (below diagonal) between populations based on 12S + CR *mt*DNA sequences of *C.* *guanhumi*.

Localities	Alagoas	Bahia	Florida	Puerto Rico	Sergipe
Alagoas	-	0.5378	47.9405 **	29.2992 **	0.0736
Bahia	0.0278 *	-	47.2802 **	28.8834 **	0.1489
Flórida	0.6649 **	0.6590 **	-	2.7508 **	48.1907 **
Puerto Rico	0.4586 **	0.4531 **	0.0690 **	-	29.4809 **
Sergipe	0.0043	0.0078	0.6725 **	0.4791 **	-

** Significant at *p* < 0.01 and * Significant at *p* < 0.10 after correction for multiple tests.

**Figure 4:**
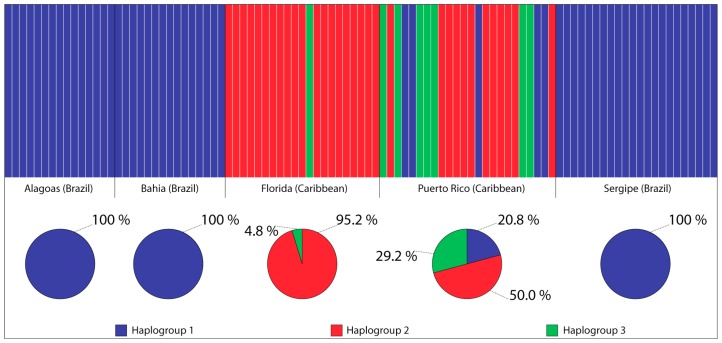
Bayesian assignment analysis for 12S + CR sequences of the five localities sampled. Each vertical bar represents an individual and its associated probability of belonging to one of the three haplogroups detected (*p* = 1).

The Bayesian Analysis of Population Structure (BAPS) detected three mitochondrial DNA haplotype clusters, hereafter referred to as haplogroups, across the five populations sampled, one of which was restricted o the Brazilian waters (HG1), with minor presence in Puerto Rico (Caribbean Sea), and two haplogroups (HG2 e HG3) restricted to the Caribbean exclusively (Figure 4). Within Brazilian waters, ALA, BAH and SER were exclusively represented by one of the haplogroups (HG1 = 100%), whilst FLO was represented by HG2 (95.2%) e HG3 (4.8%), and PUR, within the Caribbean, was represented by all three haplogroups (HG1 = 20.85; HG3 = 29.2%), but mostly by HG2 (50%). All individuals were correctly assigned to their respective haplogroups (*p* > 0.05). It is worth observing that the number of detected haplogroups (3) corresponded to the number of clades depicted in the NJ-tree and the median-joining network (Figure 2, Figure 3 and Figure 4).

**Figure 5:**
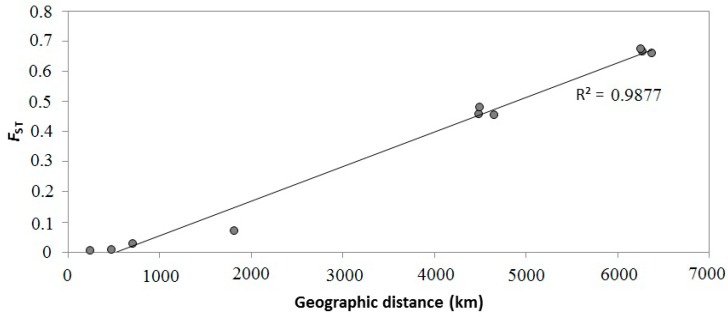
Isolation by distance (IBD) used Mantel tests (10,000 permutations) for five *C. guanhumi* populations based on *F*_ST_ as genetic distance (R^2^ = 0.987, *p* < 0.05). Each point represents one population pairwise FST plotted against geographic distance between paired populations.

By a Mantel test, a significant correlation was found to exist between pairwise population *F*_ST_ values and the geographic distances between localities (Mantel test, *p* < 0.05, Figure 5).

### 2.4. Tests of Neutrality and Estimates of Population Expansion

Results from Tajima’s D and Fu’s *Fs* tests are shown in Table 4 for each individual population. Tajima’s D, which measures the disparity between the number of segregating sites and the pairwise genetic distance, resulted in negative values for each locality, except Puerto Rico. However, Tajima’s D statistic showed values not significantly different from zero (*p* > 0.05). The Fu’s *F_S_*-test, which is devised specifically to detect population expansion and is more sensitive to the presence of singletons in the samples, showed significant values in four populations (ALA, FLO, PUR, SER), but with a mean not significant negative value (*Fs* = −0.74997; *p* > 0.10).

**Table 4:** Statistical tests for neutrality and the estimate of demographic parameters for *C. guanhumi* based on 12S + CR *mt*DNA sequence data.

	Population	Alagoas	Bahia	Florida	Puerto Rico	Sergipe	Total (All Pooled)
Parameter							
Tajima’s D (*p*-value)		−0.73010 (0.21800)	−0.53083 (0.31700)	−1.13865 (0.10800)	0.14094 (0.62100)	−0.91345 (0.16800)	−0.11210 (0.34100)
Fu’s *Fs* (*p*-value)		−4.59719 (0.01300)	−0.12846 (0.45600)	−6.12142 (0.00700)	−5.40002 (0.02400)	−6.54237 (0.01000)	−0.74997 (0.10100)
τ		13.88281	18.82812	25.78320	28.33203	15.07617	22.0266
SSD (*p*-value)		0.03089 (0.02000)	0.02758 (0.05000)	0.00878 (0.13000)	0.01667 (0.08000)	0.02560 (0.02000)	0.02909 (0.05790)
Raggedness (*p*-value)		0.01342 (0.85000)	0.05705 (0.01000)	0.00980 (0.69000)	0.00893 (0.59000)	0.01006 (0.61000)	0.00415 (0.20109)

τ, units of mutational time; SSD, sum of squared deviations; Raggedness, Harpending [37] raggedness index.

**Figure 6:**
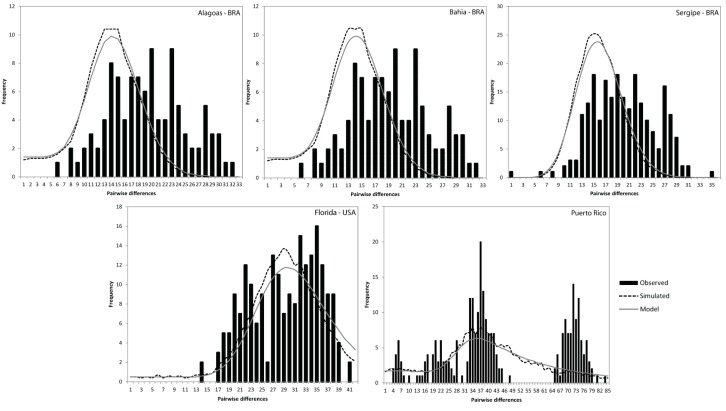
Frequency distributions of the number of pairwise nucleotide differences (mismatch) between *C. guanhumi* haplotypes for all populations.

Mismatch distributions for *C. guanhumi* individuals sampled from all five localities were constructed respectively (Figure 6). Results showed that mismatch distributions were multimodal and ragged for all the five populations. Values for the raggedness statistic, which measures the smoothness of the mismatch distribution [37], were mostly not significant, except for BAH. Despite the significant values of the sum of square deviations (SSD) for ALA, BAH and SER, total SSD was not significantly different from zero.

### 2.5. Discussion

The analysis of the contiguous *mt*DNA sequences of the 12S rRNA and CR fragments revealed considerable genetic variation in sampled localities. High values of haplotype diversity and of nucleotide diversity were observed (Table 1), which is similar to a previous report based on the same investigated species from populations distributed along the Brazilian coast using the mitochondrial DNA (*mt*DNA) control region as marker [31]. The high genetic variability observed in *C. guanhumi* is also in agreement with data from other marine organisms, particularly from other marine crustacean species with planktonic larvae using the same mitochondrial markers [38,39]. The extensively high values for haplotype and nucleotide diversity could be attributed to the complex and variable nature of *C. guanhumi* population distribution in the Western Central Atlantic [40] with deep evolutionary history, a combination of a high mutation rate in the study fragment and the large population size of the species [41,42]. This result indicates to some extent that the genetic diversity of the blue land crab is characterized by high genetic variability distributed homogeneously within each main geographical region (Caribbean Sea and the Brazilian coast), as similarly observed in a previous study within Brazilian mangroves exclusively [31], and on other crab species (e.g., [43,44]).

The patterns observed in this species are particularly comparable to those obtained for other crustacean (e.g., *P. argus*) in a study carried out using the same *mt*DNA fragment [45]. These two species, *C. guanhumi* and *P. argus*, share similar geographical ranges, from southern USA to southern Brazil. Both species do seem to experience obstacles for gene flow in the region encompassed by their studies (*i.e.*, Western Central Atlantic). The dispersal capacity of their larvae is probably diminished by the Amazon and Orinoco River plumes and North Brazil Current (NBC) rings, which may be acting as a geographical barrier.

The topology of the NJ tree was an additional indication of a distinct pattern of phylogeographic structure among the 93 haplotypes (Figure 2), suggesting that the perceived population structure is likely due to restricted contemporary gene flow. The genetic distance between the two major groups also indicates the presence of one or more barriers to dispersal that may have prevented, or be preventing, migration between these two main geographic areas. However, the occurrence of five Puerto Rican haplotypes in the Brazilian cluster suggests that migration and gene flow may also have been possible in some cases (now or at some time in the evolutionary past) between the Caribbean island of Puerto Rico and Brazilian estuaries, but not sufficient to ensure complete *mt*DNA homogenization among regions.

The reconstructed network (Figure 3) displayed a deep division into three haplogroups with no dominant *mt*DNA haplotypes in populations, but instead a distribution of a few low-frequency recurrent haplotypes with a large number of singletons. One haplogroups (HG1) was restricted to the estuaries along the Brazilian coastline, which were closely related and did cluster together, and the other two haplogroups (HG2 + HG3) were restricted to the Caribbean. This evidenced sharp genetic separation between two geographical regions might be attributed to differences in sequence divergences between haplotypes. The analysis of *mt*DNA sequence data also revealed that population samples within the Brazilian coast are nearly homogenous. The MJ network of the observed 93 haplotypes did reveal a possible geographic pattern within the Caribbean (Figure 3), despite that high levels of population homogeneity have been reported in several invertebrates species with planktonic larval phases along the Atlantic coast (e.g., *Farfantepenaeus brasiliensis* and *Litopenaeus schmitti* [46]; *Ucides cordatus* [47]; *Cardisoma guanhumi* [31]). Moreover, the hypothesis of population divergence between Caribbean Sea and Brazilian populations is also in agreement with results from analysis of population differentiation. The AMOVA test of *C. guanhumi* *mt*DNA revealed that 48.74% of the genetic variation occurred among the populations (Table 2), which might suggest a strong and stable regional genetic structure in this species. Estimated *F*_ST_-values among populations (Table 3) from the Brazilian sites (ALA, SER, BAH) showed no restricted levels of gene flow and thus suggest larval exchange between the sample sites (possibly with stepping stone populations in between). Although such lack of population differentiation is found in other species (e.g., *Pachygrapsus crassipes* [44] and *Callinectes sapidus* [43]), it is certainly not shared by all estuarine crustaceans.

Despite its relatively short planktonic larval stage (up to 65 days [48]) the mixing properties of the oceans (*i.e.*, off the Brazilian coast) seem to be responsible to a near-homogeneous diversity in the Brazilian coast (Figure 3 and Figure 4). The pelagic larval stage may still be susceptible to dispersal by water currents to increase gene flow over large extensions and to decrease population differentiation [49]. Such pattern may also be a reflection of past geological and climatic events (e.g., plate tectonic movement or glacial episodes) [50]. These presumptions may explain the genetic similarities among the estuaries along the Brazilian coast. Additionally, successive continuous migration over shorter distances between adjacent mangroves may occur to further explain connectivity within Brazilian waters [31].

The presence of this phylogeographical discontinuity was further demonstrated by the results of the Bayesian assignment analysis (Figure 4), according to which individuals assigned to haplogroups II and III were absent in the Brazilian sites. The confinement of haplogroup I individuals within the Brazilian coast may result from preferential gene flow within the Brazilian coastline.

Results from Mantel test indicate isolation by distance, which supports the hypothesis that genetic differences were caused by restricted dispersal between The Caribbean and Brazilian sampling sites (Figure 5). For semi-terrestrial estuarine species with pelagic larvae, several causes can be involved in determining a genetic population structure: local ocean currents; presence of physical barriers such as islands; life history features; recent history as well as direct control over dispersion by both adults and juveniles differential survival or mating success of immigrants after settlements [51,52]; effect of natural temporal variation in the oceanographic processes. Unfortunately, there are only incomplete data on *C. guanhumi* larval dispersal mechanisms, and more studies on the species biology, as well as on the local currents, are needed. At the present, based on our results, a simple model of isolation by distance can also explain the population genetic structure of *C. guanhumi* within the tropical Western Central Atlantic region since population genetic differentiation (estimated as *F*_ST_) does appear to be related to geographic distances separating populations (Figure 5 and Table 3).

Also in this study, we performed Tajima’s D test, Fu’s *Fs* analysis for neutral evolution, and mismatch distribution analysis to examine the historical demographic expansions of *C. guanhumi*, which may play an important role in determining the patterns of genetic variability [53,54]. The resulting negative values for Tajima’ D and Fu’s *Fs*-tests, but non-significant (Table 4), and a ragged distribution of mismatched distribution frequency (Figure 6) indicate a complex demographic history of *C. guanhumi* populations in the Western Central Atlantic, which might corresponds to bottleneck and subsequent population growth [55].

## 3. Experimental Section

### 3.1. Sampling Sites and DNA Extraction

A total of 97 specimens of *Cardisoma guanhumi* were collected from 5 mangrove habitats along the Western Central Atlantic coast: in *Vieques* Island (18°09′39.6′′ N, 65°23′21.3′′ W, Puerto Rico, N = 24); Florida (25°22′58.7′′ N, 81°11′47.4′′ W, USA, N = 21); Alagoas (09°24′51.32′′ S, 35°29′51.42′′ W, Brazil, N = 15); Bahia (14°48′27.19′′ S, 39°02′00.13′′ W, Brazil, N = 15); Sergipe (10°58′33.8′′ S, 37°03′13.9′′ W, Brazil, N = 22) (Figure 1). Muscle tissue samples were taken in the field from a portion of the walking leg (pereiopods) from each individual, stored in 100% ethanol and shipped to the laboratory for molecular analysis. Total genomic DNA was extracted from the muscle tissue using proteinase K and phenol-chloroform extraction [56], resuspended in Tris-EDTA (TE) buffer (pH 7.5). High molecular weight DNA was quantified by gel electrophoresis (1% agarose gel) staining comparison.

### 3.2. Mitochondrial DNA Amplification and Sequencing

The largest portion of the control region and a 112-bp short fragment of the 12S gene within *C. guanhumi* *mt* genome was amplified by a pair of primers, DLUSSAF (5′-GTA TAA CCG CGA ATG CTG GCA C-3′) and ILEUCAR (5′-CCT TTT AAA TCA GGC ACT ATA-3′ [47]). Amplification reactions were conducted in 25 μL volumes containing approx. 100 ng of template DNA, 2.5 μL of 10× PCR buffer, 1.5 mM of MgCl_2_, 200 μM of each dNTP, 0.5 μM of each primer, and 0.625 U of *Taq* DNA polymerase (New England Bio Labs, Ipswich, MA, USA). The PCR consisted of 30 cycles of 95 °C for 30 s, 50 °C for 30 s, and 68 °C for 60 s, with an initial denaturation of 95 °C for 30 s and a final extension of 68 °C for 5 min using a Veriti 96-well Thermal Cycler (Applied Biosystems, Foster City, CA, USA). All amplifications were run against a negative control in which all components, except DNA template were added. Amplicons were checked for correct size and quality by gel electrophoresis with SyberSafe™ staining.

PCR products were purified with Invitek^®^ PCR Purification columns in order to remove the excess primers and nucleotides, and concentrate the amplified PCR fragments. The purified double-stranded amplification products were used as template DNA in sequencing reactions. Cycle sequencing was performed using the ABI Prism BigDye™ Ready Mix (Applied Biosystems) and primers DLUSSAF and ILEUCAR, separately, on a Veriti 96-well Thermal Cycler. Then, sequencing products were purified using isopropanol to remove unreacted fluorescent BigDye Terminators. The PCR products were sequenced on the ABI 3500 Genetic Analyzer (Applied Biosystems) in both forward and reverse directions. All new distinct nucleotide sequences were submitted to the GenBank database (National Center for Biotechnology Information, Bethesda, MD, USA).

### 3.3. Sequence Data Analysis

Forward and reverse DNA sequences for each individual were inspected with sequence editor CHROMAS version 2.23 (Technelysium, Brisbane, QLD, Australia) and corrected when necessary. Homologous nucleotide sequences from all samples were aligned using the program CLUSTALX version 1.81 [57] on the sequence alignment editor BIOEDIT version 7.2.5 [58]. Alignments were double-checked by eye, and refined manually if needed.

The occurrence of substitution saturation, which could create a bias in phylogenetic reconstruction, was investigated by comparing half of the theoretical saturation index expected when assuming full saturation (*ISS.c*, critical value) with the observed saturation index (*ISS*) [36]. Indices of substitution saturation and entropy (Hx), a measure of the amount of variability through haplotypes sequences [58], were calculated with the program DAMBE version 5.2 [59].

Unique haplotypes were identified using DNASP version 5.10 [60]. Genetic diversity indices were estimated by computing the mean nucleotide composition, number of transitions, transversions, indels, polymorphic sites (Nps), number of segregating sites (S), haplotype (H) and nucleotide (π) diversities [61], and mean number of pairwise differences (M), all using the same software.

The aligned *C. guanhumi* *mt*DNA sequences were also imported into MEGA version 6.0 [62] for estimation of nucleotide sequence divergence between haplotypes and sampling groups under the Kimura 2-parameter model [63]. Sites with alignment gaps and missing data were omitted from the analyses. A neighbor-joining (NJ) analysis based on the matrix of Kimura 2-parameter distances was additionally performed to examine the relationships among individuals. To evaluate significance levels and the consistency of nodes (tree topology) derived from the phylogenetic analysis, 1000 bootstrap replications of the original data set were performed [64].

A network was generated to infer the genealogical relationships among haplotypes using the median-joining algorithm [65], implemented in NETWORK version 4.6.1 (Fluxus Technology Ltd, Suffolk, England, UK).

Geographical structuring of *mt*DNA variation was examined by a hierarchical analysis of molecular variance (AMOVA [66]) and population pairwise *F*_ST_ values between populations were calculated in ARLEQUIN version 3.51 [67] using the method of Weir & Cockerham [68]. Fixation indices significantly different from zero were identified by comparison with the results of 10,000 data permutations [69].

A corrected average pairwise difference (D_A_) between populations was also calculated as

D_A_ = P_i_XY − (P_i_X + P_i_Y)/2 (1)

where P_i_XY is the average of pairwise differences between populations X and Y, and P_i_X/P_i_Y are the averages of pairwise differences among individuals within population X and Y, respectively. Post-hoc Bonferroni’s correction for multiple comparisons were used when appropriate.

Additionally, a Bayesian Analysis of Population Structure (BAPS [70]) was employed to investigate the genetic structure of the species by clustering genetically similar individuals into panmictic groups. Population clusters were tested by five replicates for each value of *k* (*k* is the maximum number of clusters) up to *k* = 10, and the results were averaged according to the resultant likelihood scores.

The isolation-by-distance hypothesis was examined by a Mantel’s test [71,72]. Geographical distances were calculated by measuring the linear map distance along the coastline between each two sampling sites.

Demographic patterns of populations were assessed in DNAsp by, firstly, testing selective neutrality for each population and for all individuals using Tajima’s *D* [73] and Fu’s *F_S_* [74] statistics based on 10,000 replicates, and secondly, by plotting the observed and expected distributions of the number of pairwise mutational differences between haplotypes to their relative frequencies (mismatch distributions) under the sudden expansion model [75,76]. The Harpending raggedness index (r [37]) was used to test the fit between the observed and the expected distributions, and the sum of squared deviations (SSD) of the test of goodness-of-fit with 10,000 bootstrap replicates, as implemented in ARLEQUIN version 3.51 [67].

## 4. Conclusions

Overall, the data presented here, based on *mt*DNA CR gene sequences, identified high genetic diversity and significant genetic differentiation in *C. guanhumi* within the Western Central Atlantic. A sharp genetic break between Caribbean and Brazilian populations, which appears to be associated with a marine biogeographic boundary to gene flow, raised concerns over the conservation status of the blue land crab. The population structure implied that a clear subdivision exists in *C. guanhumi* regional populations, and should be considered as different management units for further effective conservation and management purposes [42,77]. Therefore, management strategies should be undertaken to protect this species and prevent the loss of the rich genetic variation of natural populations as well as total numbers of individuals. However, we examined only a portion (partial 12S rRNA and CR concatenated sequences) of the entire *mt* genome in this study. The use of multiple genetic marker systems could increase the resolving power of this genetic study and the inclusion of crabs from different estuaries and larger numbers of individuals at each site should confirm the pattern of genetic population structure revealed in this study. Further studies including nuclear markers (e.g., microsatellite DNA loci [18]) are needed to extend and corroborate the present population structure findings to further understand the comprehensive population structure in this crab species.
